# Prevalence and characteristics of neuropsychiatric symptoms, quality of life and psychotropics in people with acquired brain injury in long‐term care

**DOI:** 10.1111/jan.14156

**Published:** 2019-08-05

**Authors:** Roy Kohnen, Jan Lavrijsen, Odile Smals, Debby Gerritsen, Raymond Koopmans

**Affiliations:** ^1^ Vivent, Rosmalen and Livio Enschede The Netherlands; ^2^ Department of Primary and Community Care, Radboud University Medical Center Radboud Institute for Health Sciences Nijmegen The Netherlands; ^3^ Vivent Rosmalen The Netherlands; ^4^ Department of Primary and Community Care, Radboud University Medical Center, De Waalboog, “Joachim and Anna” Centre for Specialized Geriatric Care Nijmegen The Netherlands

**Keywords:** acquired brain injury, long‐term care, neuropsychiatric symptoms, nurses/midwives/nursing, nursing home, prevalence, psychotropic drugs, quality of life

## Abstract

**Aim:**

Establishing the prevalence of neuropsychiatric symptoms (NPS), quality of life and psychotropic drug use in people aged ≤65 years with acquired brain injury in nursing homes.

**Design:**

Cross‐sectional, observational study among patients aged 18–≤65 years with acquired brain injury admitted to special care units in Dutch nursing homes.

**Methods:**

According to the Committee on Research Involving Human Subjects in January 2017 this study did not require ethics approval. Nursing homes will be recruited through the national acquired brain injury expertise network for patients with severe brain injury, the regional brain injury teams and by searching the internet. Patient characteristics will be collected through digital questionnaires. Neuropsychiatric symptoms will be assessed with the NeuroPsychiatric Inventory‐Nursing Home version, the Cohen–Mansfield Agitation Inventory and the St. Andrews Sexual Behaviour Assessment; cognition with the Mini‐Mental State Examination, quality of life with the Quality of Life after Brain Injury Overall Scale and activities of daily living with the Disability Rating Scale. Medication will be retrieved from the electronic prescription system. Data collection commenced in 2017 and will be followed by data analysis in 2019. Reporting will be completed in 2020.

**Discussion:**

Little is known about NPS among patients with acquired brain injury in nursing homes. In patients up to the age of 65 years, only six studies were found on prevalence rates of NPS.

**Impact:**

Patients with severe acquired brain injury experience lifelong consequences, that have a high impact on them and their environment. Although there is increasing attention for the survival of this vulnerable group of patients, it is also important to enlarge awareness on long‐term consequences, specifically the NPS, quality of life and psychotropic drug use in acquired brain injury. Insight into the magnitude of these issues is necessary to achieve appropriate care for these patients.

## Abstract (zh)

**目的:**

确定神经精神病症状的患病率(NPS)、住在疗养院的年龄≤65 岁的脑损伤老年人的生活质量和精神药物。

**设计:**

针对疗养院内年龄在18‐–≤65 岁的特殊护理病房的脑损伤病人的横段截面观察性研究。

**方法:**

根据人体研究委员会在2017年一月的报告,本研究不需要道德批准。疗养院将通过数据问卷调查收集病人的特征。神经疾病将根据神经精神病学目录‐疗养院版本、Cohen–Mansfield精神激动目录和St. Andrews的性行为评估评估神经精神疾病、认识精神状态检查量表脑损伤后总体评分和残疾评定量表。电子处方系统为开具药物。我们在2017年开始数据收集,并且将在2019 年进行数据分析,2020年完成报告。

**讨论:**

针对疗养院中脑损伤病人患有神经精神疾病的研究甚少。仅有6项研究针对的是65岁老年人的神经精神疾病患病率。

**影响:**

严重脑损伤可能会影响病人的一生,不仅对其自身也会其周围环境产生严重的后果。尽管有越来越多的人关注弱势病人群体的生存问题。但也有必要提高其对长期后果的认识,尤其是NPS、生活质量、对后天脑损伤的神经精神药物治疗。深入了解这些疾病的也是为病人提供正确治疗所必要的步骤。

## 1 INTRODUCTION

Acquired brain injury (ABI) is an injury to the brain that is not hereditary, congenital, degenerative or induced by a birth trauma (Brain Injury Association of America, 2019). ABI can be traumatic or non‐traumatic and the damage can be focal or diffuse. Brain injury is a major cause of death and disability (Fakhry, Trask, Waller, & Watts, 2004). Most injuries occur in the very young (0–4 years), adolescents (15–24 years) and in people over 65 years of age (Brown, Elovic, Kothari, Flanagan, & Kwasnica, 2008). In a Dutch study (*N* = 1892), accidents appeared to be the most common cause of traumatic brain injury (TBI) as were hypoxic‐ischaemic events for non‐TBI in youth aged 1 month to 24 years (Kloet et al., 2013). The consequences can range from a mild temporary concussion to severe damage or death. Severe damage may result in more permanent disorders of consciousness including unresponsive wakefulness syndrome (UWS), formerly known as vegetative state (VS) and the minimally conscious state (MCS; Giacino et al., 2002; Jennett & Plum, 1972; Laureys et al., 2010).

Patients who emerge from disorders of consciousness may have cognitive deficits, such as impaired long‐term memory, executive function and self‐awareness (Azouvi, Arnould, Dromer, & Vallat‐Azouvi, 2017). Executive functioning deficits are common following TBI and both cognitive and behavioural functions fall under the general umbrella of executive functioning (Rabinowitz & Levin, 2014). They are a variety of cognitive abilities carried out predominantly by regions in the prefrontal cortex and many of these can be affected by TBI. Behavioural changes may be related to lack of control like disinhibition, impulsivity, irritability, aggression, or lack of drive such as apathy, reduced initiative, poor motivation (Azouvi et al., 2017). Neuropsychiatric symptoms (NPS), such as aggression, socially inappropriate behaviour and disinhibition, may occur (Carlier & Kramer, 2006; Rasquin & Van Heugten, 2007). A study of 120 patients aged 18–65 years and 45 patients over 65 years admitted to a hospital after TBI showed a significant association in both age groups between the presence of four or more NPS and an impaired cognitive state (Deb & Burns, 2007). According to another study of 196 hospitalized patients 18–94 years of age one year after TBI, lower MMSE score seemed to be an important risk factor in the development of a psychiatric illness, such as depressive and panic disorder (Deb, Lyons, Koutzoukis, Ali, & McCarthy, 1999).

### 1.1 Background

Neuropsychiatric symptoms put a high burden on patients and their environment such as family and nursing staff (Alderman, 2007; Johnson & Balleny, 1996). NPS such as aggressive behaviour and inappropriate sexual behaviour (ISB) amongst patients with ABI are problematic for patients, families and professionals (Alderman, Knight, & Henman, 2002; Knight et al., 2008). Although ISBs were present in a minority of patients with severe TBI (8.9% of *N* = 507) and a mean age of 32.7 years at injury in a community‐based rehabilitation cohort, they pose a complex clinical challenge (Simpson, Sabaz, & Daher, 2013). In many cases ISBs were accompanied by other NPS, most often inappropriate social behaviour and/or aggression.

One study conducted in a specialized postacute treatment centre for adult inpatients with ABI, which is part of a large general psychiatric hospital, (*N* = 57; mean age 49.2 years) found significant associations of aggression with gender, legal status on admission (voluntarily or involuntarily), duration of admission and hypoxia as a cause of ABI (Visscher, van Meijel, Stolker, Wiersma, & Nijman, 2011). The Dutch version of the Staff Observation Aggression Scale‐Revised was used to document the prevalence, nature and severity of aggression incidents, which is widely used in general psychiatric institutions for monitoring both verbal and physical aggression against objects, patients, staff or others (Nijman, 2002; Visscher et al., 2011). Male patients were significantly more aggressive and patients who were involuntarily admitted were substantially more likely to display aggression. The duration of admission was significantly longer, 2.5 years on average, for patients with aggression compared with patients without aggression. Of the nine patients with hypoxia as aetiology, seven were aggressive.

Neuropsychiatric symptoms can pose considerable challenges to quality of life (QoL) in TBI (Warriner & Velikonja, 2006). Quality of life was described with the Quality of Life Inventory developed by Frisch in a sample of adults with TBI (*N* = 50; mean age 38.74 years) living in the community (Frisch, 1994; Kalpakjian, Lam, Toussaint, & Merbitz, 2004). The mean QoL in these patients was low (mean 43.08; range 3–71) compared with non‐injured adults and low levels of QoL reflect unhappiness and a lack of fulfilment, with limited resources to meet the demands of life. Quality of life was established in three studies of 157, 126 and 795 patients, respectively with (traumatic) brain injury (mean age 43.1 and 39 years) from (residential) rehabilitation and trauma centres (Siponkoski, Wilson, Steinbuchel, Sarajuuri, & Koskinen, 2013; Soberg et al., 2013; von Steinbuchel et al., 2010). Lower QoL, established with the Quality of Life after Brain Injury (QOLIBRI) questionnaire, was related to depression and anxiety. Another study using the QOLIBRI in patients with TBI (*N* = 504; mean age 42 years) discharged to home, showed that the only direct predictors of lower QoL were mood and cognition (Azouvi et al., 2016).

Neuropsychiatric symptoms may prompt prescription of psychotropic drugs in an urgent need to control behaviour to prevent harm and allow safer and more effective management of the patient (Hammond et al., 2015; Mysiw et al., 2006). In long‐term care, tranquillizers (59%) are the most prevalent psychotropic drugs followed by anticonvulsants (35%) and antidepressants (26%–34%; Kohnen, Gerritsen, Smals, Lavrijsen, & Koopmans, 2018). According to a survey of 168 psychiatrists of which 49 were available for analysis, there was limited uniformity in drug selection for the various NPS (Francisco, Walker, Zasler, & Bouffard, 2007). This was potentially due to the availability of various medications with a similar effect, the variability of clinical presentation and severity of the NPS and the lack of clinical practice guidelines. Antipsychotics, which are prescribed for the treatment of psychosis, agitation and aggression, may have adverse effects on cognition and severe side effects in the long‐term such as stroke and increased mortality (Ballard, Corbett, Chitramohan, & Aarsland, 2009; Stanislav, 1997). Cognitive behavioural therapy, a non‐pharmacological anger self‐management technique, appears to be a safe and effective tool to control aggression in a population with ABI as alternative to medication use (Iruthayarajah et al., 2018).

Patients with ABI who are unable to live at home, for example because of NPS, are commonly admitted to long‐term care facilities (LTCF), mainly nursing homes. Long‐term care refers to health, social and residential services given to chronically disabled persons over an extended period of time (Doty, Liu, & Wiener, 1985). In the long‐term care population of patients with ABI below 65 years of age, depressive symptoms are most common with a prevalence ranging from 13.9% to 39.3% followed by socially inappropriate behaviour (16%–25.2%), physically abusive behaviours (7.8–18%) and anxiety (2.8–10%; Kohnen et al., 2018).

Little is known about the population of patients ≤65 years of age with ABI residing in nursing homes. The conclusion from a recent systematic review is that in patients up to the age of 65 years with ABI in long‐term care, only six studies were found about prevalence rates of NPS in long‐term care and two of these studies reported prevalence rates of psychotropic drug use (PDU; Kohnen et al., 2018). More insight into the magnitude of NPS and PDU is necessary to achieve appropriate care, such as enhancing uniformity in drug selection, limiting PDU and promoting non‐pharmacological interventions, for patients with ABI ≤65 years of age in long‐term care.

## 2 THE STUDY

### 2.1 Aim

The aim of this study is to establish the prevalence and characteristics of: (a) NPS in general; (b) aggression and ISB in particular; (c) psychotropic drug use; (d) the impact of NPS on quality of life; and (e) the determinants of the behaviours among patients with ABI ≤65 years of age in Dutch nursing homes.

### 2.2 Timescale

Data collection commenced in 2017 and will be followed by data analysis in 2019. Reporting will be completed in 2020.

## 3 METHODS

### 3.1 Study design

The CABINET‐study (Chronic Acquired Brain Injury Netherlands) is a cross‐sectional, observational study among people 18–≤65 years of age with chronic ABI in Dutch nursing homes.

### 3.2 Procedure

#### 3.2.1 Recruitment nursing homes

For logistical reasons, nursing homes with special ABI care wards for at least 10 ABI patients will be identified, approached and recruited through: (a) the national expertise network for patients with severe ABI (EENnacoma, 2017); (b) the regional brain injury teams; and (c) the internet sites of long‐term care organizations. In the Netherlands, 17 regional brain injury teams throughout the country advice and give information about ABI to patients with ABI, family members and professional caregivers (Hersenletselteams (Brain Injury Teams) (2019)). Nursing homes which have participated in previous prevalence studies will be approached and recruited as well (van Erp et al., 2015; Kohnen, Lavrijsen, Bor, & Koopmans, 2013; Lavrijsen, van den Bosch, Koopmans, & van Weel, 2005). At recruitment, all relevant study documents, such as the assessment instruments, will be sent to the nursing home's science committee for review or the treating physician if a science committee is not present. To achieve an optimal response rate, the researcher will organize local instruction meetings to explain the purpose and the goals of this study.

#### 3.2.2 Residents

An e‐mail will be sent to the treating physicians of the identified nursing homes to inform them about the study and to ask them to systematically screen all residents ≤65 years of age in the chronic stage of ABI for inclusion. Inclusion criteria are: (a) nursing home admission because of ABI; (b) in the chronic phase of non‐progressive forms of ABI; (c) age from 18 up to and including 65 years of age; and (d) residing in the nursing home for at least 4 weeks at the time of inclusion. The exclusion criteria are: (a) nursing home admission other than ABI; (b) progressive forms of ABI such as multiple sclerosis; (c) admitted for rehabilitation, temporary admission, or outreaching nursing home care; (d) disorders of consciousness such as the UWS; and (e) being terminally ill at the time of inclusion. The inclusion and exclusion criteria are listed in more detail in Table 1.

**Table 1:** Inclusion and exclusion criteria

Inclusion	Exclusion
Nursing home admission because of ABI	Nursing home admission other than ABI
Causes of ABI	Causes of ABI:
Traumatic: traffic, falling, violence, sports	Progressive degenerative: dementia, multiple sclerosis, Parkinson's disease, Huntington's disease, Korsakoff's syndrome, progressive supranuclear palsy, mitochondrial disease, cerebellar ataxia, multisystem atrophy, stroke in progressive or degenerative disorder, brain tumour
Non‐traumatic: stroke, post brain tumour, anoxia, subarachnoid haemorrhage, cerebral infections, intoxications, endocrine disorder, feeding deficits	
Age of 18− ≤65 years at time of inclusion	
Chronic phase after ABI	Rehabilitation, temporary admission, outreaching nursing home care
	Disorders of consciousness: coma, unresponsive wakefulness syndrome, minimally conscious state
	Terminally ill at the time of inclusion, life expectancy less than 3 months
Reside in the nursing home for at least 4 weeks at the time of inclusion	

#### 3.2.3 Measurements

The instruments to assess the NPS, cognition, activities of daily living (ADL) and quality of life are listed in Table 2.

**Table 2:** The instruments to assess the neuropsychiatric symptoms (NPS), cognition, activities of daily living, and quality of life

Instrument	Description
Neuropsychiatric Inventory‐Nursing Home Version (NPI‐NH)	Structured interview to assess 12 NPS: delusions, hallucinations, agitation, depression, anxiety, euphoria, apathy disinhibition, irritability, aberrant motor behaviour, night‐time disturbances, and appetite/eating change. Score ranges from 0 to 144. A higher score represents more severe symptoms
Cohen–Mansfield Agitation Inventory (CMAI)	Instrument to assess 29 agitated or aggressive behaviours
St. Andrews Sexual Behaviour Assessment (SASBA)	Instrument to establish inappropriate sexual behaviour in progressive and acquired neurological impairment, consisting of four categories: verbal comments, non‐contact, exposure, and touching others with severity levels ranging 1–4. A higher score represents more severe behaviour. Antecedents are assessed by 25 factors and the interventions by 14 items
Mini‐Mental State Examination (MMSE)	Includes 11 questions and measures cognitive functions: orientation, attraction, concentration, memory, language and constructive capacity
Disability Rating Scale (DRS)	Instrument consisting of eight sections: eye opening, communication ability, motor response, feeding, toileting, grooming, level of functioning, and employability. Total score ranges between 0 and 30. A higher score represents a higher level of disability
Quality Of Life After Brain Injury Overall Scale (QOLIBRI‐OS)	Six questions covering physical condition, cognition, emotions, function in daily life, personal and social life, and current situation and future prospects

#### 3.2.4 Assessment of NPS

The professional caregivers involved in the daily care of the residents will observe symptoms over 2 weeks prior to assessment. After this period, these professional caregivers will be visited by the first author or the research assistant for a structured interview. They will also be asked to fill in assessment instruments through a web‐based digital system which will be sent by e‐mail. The use of these web‐based instruments will be explained in the local instruction meetings.

Neuropsychiatric symptoms will be assessed with the Neuropsychiatric Inventory‐Nursing Home Version (NPI‐NH; Cummings, 1997; Cummings et al., 1994). The NPI‐NH was originally developed for dementia and is a structured interview including 12 NPS: delusions, hallucinations, agitation, depression, anxiety, euphoria, apathy, disinhibition, irritability, aberrant motor behaviour, night‐time disturbances and appetite/eating change. The frequency (F) and the severity (S) of each symptom are rated on a four‐ (1–4) and three‐point (1–3) Likert scale based on the structured questions administered to the patients’ professional caregiver. A score can be calculated for each symptom by multiplying the frequency and the severity (F×S scores) resulting in values ranging from 0 to 12. The sum of the 12 FxS scores leads to a total score ranging from 0 to 144. A higher score represents more severe symptoms. NPS are considered clinically relevant when the FxS score for each item is 4 or more. The nursing home version, developed for use by professional caregivers in institutions, has been translated into Dutch and found valid and reliable for trained nursing home staff (Kat et al., 2002; Wood et al., 2000). The NPI has been used in several studies on TBI and stroke and is considered suitable for assessing NPS in ABI (Angelelli et al., 2004; Castano Monsalve, Bernabeu Guitart, Lopez, Bulbena Vilasar, & Ignacio Quemada, 2012; Castellanos‐Pinedo et al., 2011; Ciurli, Formisano, Bivona, Cantagallo, & Angelelli, 2011; Kilmer et al., 2006; Rush et al., 2010).

Aggression will be assessed using the Cohen–Mansfield Agitation Inventory (CMAI; Cohen‐Mansfield, 1986). This instrument is the most widely used assessment scale for measuring the frequency of agitation and aggression and defines these behaviours as inappropriate verbal, vocal or motor activities not explained by apparent needs or confusion. This instrument assesses 29 agitated or aggressive behaviours which can be categorized into three subscales: (a) physically aggressive (directed against a person or object); (b) physically non‐aggressive (not directed against a person or object, such as pacing and wandering); and (c) verbally agitated behaviour. Items are scored on a seven‐point frequency scale: 1 = never; 2 = <once a week; 3 = 1–2 times a week; 4 = several times a week; 5 = 1–2 times a day; 6 = several times a day; 7 = several times per hr. Aggression is considered as clinically relevant when the behaviour appears at least once a week or more (frequency score of three or more). The CMAI has been validated in the assessment of behavioural disorders in elderly nursing home patients (Cohen‐Mansfield, 1986; Miller, Snowdon, & Vaughan, 1995). A Dutch translation is available and has been validated in elderly patients admitted to a psychiatric hospital (de Jonghe and Kat (1996)). As far as we know, this is the first time that the CMAI is used in ABI.

ISB will be assessed by the Dutch version of the St. Andrews Sexual Behaviour Assessment (SASBA; Bartelet, Waterink, & van Hooren, 2014; Knight et al., 2008). The scale consists of four sexual behaviour categories, verbal comments, non‐contact (e.g. making obscene gestures), exposure and touching others, with each four severity levels ranging from mild (for example blowing kisses or staring at another person's breasts) to severe (for example masturbating with genitals being clearly exposed in a public setting) (Bartelet et al., 2014). Each item is rated on a six point Likert scale (0–5): never; happened once; happened less than once a month; happened less than once a week; happened every week; or happened several times a week. The total score of the scale ranges from 0 to 80, a higher score representing more severe ISB. The original SASBA was designed to establish ISB in progressive and acquired neurological impairment and has strong construct and content validity and good inter‐rater and test–retest reliability (Knight et al., 2008).

#### 3.2.5 Assessment of ADL

The Disability Rating Scale (DRS) will be used to describe and assess ADL disabilities. The DRS consists of eight sections: eye opening, communication ability, motor response, feeding, toileting, grooming, level of functioning and employability. Each item is rated on a four‐, five‐, or six point Likert scale. Communication ability, which is of specific interest in this study, is rated on a 5‐point scale: 0 = oriented (e.g., patient is able to tell who he is); 1 = confused (e.g., responses are delayed); 2 = inappropriate (e.g., speech in an exclamatory way, such as shouting); 3 = incomprehensible (e.g., moaning or groaning); 4 = none (no sounds of communications signs from the patient). The total DRS score ranges between 0‐29, a higher score representing a higher level of disability. The DRS was originally developed and tested in severe head trauma patients (Rappaport, Hall, Hopkins, Belleza, & Cope, 1982). It has been recommended as one of the most appropriate instruments to assess long‐term outcomes in severe brain damage (Bullock et al., 2002; Eilander et al., 2007). The DRS has been translated into Dutch, adapted to be filled out by a proxy of the patient and used in severe ABI (Eilander et al., 2007).

#### 3.2.6 Assessment of cognition

Cognitive functioning will be assessed with the Mini‐Mental State Examination (MMSE; Folstein, Folstein, & McHugh, 1975). The MMSE includes 11 questions and measures orientation, attraction, concentration, memory, language and constructive capacity. The total score ranges from 0 to 30, a lower score represents lower cognitive functioning. A Turkish study validated the use of a Turkish version of the MMSE in ABI and found that the MMSE can be used as a cognitive screening tool in this group (Elhan et al., 2005). The MMSE has been used in several TBI studies (de Guise et al., 2011, 2013).

#### 3.2.7 Assessment of quality of life

The Dutch version of the QOLIBRI Overall scale will be used to assess QoL in patients with ABI (von Steinbuechel et al., 2012). The overall scale, a short version of the QOLIBRI, consists of six questions with regard to satisfaction with life after brain injury. Areas covered are physical condition, cognition, emotions, function in daily life, personal and social life and current situation and future prospects. Each question can be answered on a five‐point Likert scale (1–5) ranging from ‘not at all satisfied’ to ‘very satisfied’. The total score is divided by the actual number of answered questions giving a scale mean (1–5). The scale means are converted to a 0–100 percentage scale by subtracting 1 from the mean and then multiplying by 25 (QOLIBRI, 2018). A score of 0 represents the lowest and 100 the best QoL. The scale has been found a valid and reliable scale that can be used as a brief index of health‐related quality of life in TBI (von Steinbuechel et al., 2012).

#### 3.2.8 Assessment of patient characteristics

The treating physicians will be asked to digitally register the patient characteristics listed in Table 3 with the use of a web‐based questionnaire.

**Table 3:** Patient characteristics

Characteristics	Description
Sex	
Date of birth	
Marital status	Single, married, divorced, widowed
Level of education	Highest level of education at the moment of brain injury
Cause of ABI	Traumatic
	Non‐traumatic: stroke, post brain tumour, anoxia, subarachnoid haemorrhage, cerebral infections, intoxications, endocrine disorder, feeding deficits
Date of brain incident	
Date of admission in nursing home	
Place of residence before admission	Home, hospital, rehabilitation centre, institution for mentally disable persons, institution for physically disabled persons, rehabilitation ward in nursing home, another nursing home, mental health institution
Psychiatric history before brain injury	
Bladder management	Urinary catheter
Airway management	Tracheostomy or tracheal cannula with/without mechanical ventilation
Feeding	Nasogastric feeding tube or a percutaneous endoscopic gastrostomy (PEG)
Complications	Presence of delirium, pain, urinary retention, constipation, spasms at reference moment

The presence of pain, urinary retention, constipation, spasms and delirium will be described in a dichotomous manner (yes/no) at reference moment. Pain, urinary retention, constipation and spasms are physical consequences of ABI possibly due to neurological damage (Bracci et al., 2007; Hersenstichting (Brain Foundation Netherlands), 2014; Stocchetti & Zanier, 2016). Delirium has been described in TBI (Colantonio, Hsueh, Petgrave, Hirdes, & Berg, 2015; Gion & Leclaire‐Thoma, 2014). Brain injuries, stroke and the use of sedatives among others are precipitating factors for a delirium. According to the Dutch guideline Multidisciplinary Approach of Problem Behaviour, all of these complications may lead to NPS (Verenso, 2008).

#### 3.2.9 Psychotropic drug use

The data on psychotropic medication (name, dosage, continuous and/or incidental usage and prescription reason) will be retrieved from the electronic prescription system. The treating physician will be asked to register the indications for PDU (e.g., depression, anxiety, epilepsy, or neuropathic pain) to determine if patients actually receive psychotropic drugs because of NPS. Medications will be classified using the Anatomical Chemical classification (WHO Collaborating Centre for Drug Statistics Methodology, ). The psychotropic drugs will be grouped into antipsychotics, anxiolytics, hypnotics, antidepressants, anticonvulsants.

### 3.3 Statistical analysis

All data will be analysed with the Statistical Package for Social Science. Descriptive statistics will be used to describe the patient characteristics as shown in Table 3.

The frequency of each clinically relevant NPS from the NPI‐NH, the 29 behaviours from the CMAI and ISB from the SASBA will be described in numbers and percentage of patients who have these behaviours. With regard to PDU, the names of the prescribed drugs, usage and the prescription reasons will be presented as frequencies. Total daily dosage in milligrams will be presented as a mean with a range if medication was prescribed continuously. Also, the total scores on the MMSE, DRS and QOLIBRI‐OS will be presented as a mean with a range.

Analyses will be conducted with the NPI‐NH, the aggression subscales of the CMAI, the SASBA and PDU as dependent variables. Also, we will analyse whether NPS and PDU are associated with QOLIBRI‐OS. MMSE and DRS are included as independent variables. The independent Student's *t* tests or χ^2^ (chi‐square) test will be used to analyse possible differences in subgroups, specifically gender, age, marital status, level of education, cause of ABI and presence of complications. Univariate analysis will be used to identify possible determinants of NPS and PDU and all determinants will then be tested in multivariate regression analyses to determine their individual contribution to NPS and PDU.

According to literature, the prevalence rate of NPS in nursing homes is approximately 35%.(McMillan & Laurie, 2004) We assume that 50% of the patients would meet the inclusion criteria and that the response rate would be 50%. Through the national expertise network for patients with severe ABI, the regional brain injury teams and the nursing homes themselves, the number of patients residing in ABI special care units (*N* = 937) was retrieved. The expected population would be approximately 230 patients with ABI. According to Peduzzi, a rule of thumb is that a determinant can be studied for every ten patients (Peduzzi, Concato, Kemper, Holford, & Feinstein, 1996). With an estimated NPS prevalence rate of 35%, the number of patients with NPS would be 80 in a study population of 230 patients. The number of determinants that could be studied would be eight. However, for logistic regression this rule can be relaxed to 5–9 events per determinant (Vittinghoff & McCulloch, 2007). In that case, the minimum number of patients needed to study eight determinants would be 115. The number of NPS events in a study population of 115 patients would be 40.

### 3.4 Validity and reliability

The psychometric properties of the used instruments have been described above.

### 3.5 Ethics approval

This study (case number 2017–3143) was presented for medical ethics review at the Committee on Research Involving Human Subjects (CMO) of the district Arnhem‐Nijmegen, the Netherlands. The conclusion of the CMO in January 2017 was that it did not require ethics approval because our study does not involve scientific research according to the criteria of the Dutch Medical Research Involving Human Subjects Act (WMO) and can be conducted without review by the CMO. The research project will be performed according to the principles of the Declaration of Helsinki (World Medical Association, 2013). Patients are only included after written informed consent is given by themselves or by the legal representative if the patient is not mentally competent.

## 4 DISCUSSION

To our knowledge, this is the first study which profoundly focuses on the prevalence and characteristics of NPS, QoL and PDU in people aged 18–≤65 years with ABI residing on ABI wards in nursing homes. The circumstances in the Netherlands seem to give a good opportunity to conduct prevalence studies on consequences in ABI, given the high responses between 91% and 100% in prevalence studies of specific subcategories of ABI, VS/UWS and the Locked‐in Syndrome (LIS; van Erp et al., 2015; Kohnen et al., 2013; Lavrijsen et al., 2005). In the Netherlands, a strong academic infrastructure has been developed with academic networks and knowledge centres for specific patient categories in long‐term care where key elements are: (a) significant contribution in the medical curriculum; (b) a specialty elderly care medicine with a 3‐year specialist training programme, (c) and academic networks that provide an infrastructure for teaching, research and best practices (Koopmans, Pellegrom, & van Geer, 2017). In addition, in 2016 a national expertise network for patients with severe ABI, where nursing homes are participating and collaborating with researchers, has been established for specific subcategories of ABI, such as patients who experience long‐term consequences (EENnacoma, 2017). The goal of this national expertise network is to give an infrastructure for teaching, research and best practices for these specific groups of patients and participating nursing homes will probably be inclined to take part in this study.

### 4.1 Limitations

This descriptive study, where questionnaires are used to describe the population, may have some limitations. First, only a small number of the assessment instruments have been specifically developed or validated for the consequences, such as NPS, in patients with ABI. One of the assessment instruments which has not been validated for use in ABI is the CMAI, which might be a limitation. The Overt Aggression Scale (OAS) however, is used to assess aggressive behaviour in patients with TBI (Baguley, Cooper, & Felmingham, 2006). The original OAS has been revised into summative and retrospective versions because of completing difficulties, such as multiple incidents in a short period leading to multiple AOS reports (Giles & Mohr, 2007; Kay, Wolkenfeld, & Murrill, 1988; Sorgi, Ratey, Knoedler, Markert, & Reichman, 1991). A Dutch translation of one of these modified versions, the Modified OAS, is available but has not been validated (Buitelaar, 2019). A disadvantage of the modified versions of the OAS is that they eliminated the ability to give a rich description of individual aggressive behaviours in contrast to the CMAI (Cohen‐Mansfield, 1986; Giles & Mohr, 2007). Another disadvantage is that the use of the OAS requires training (Castano Monsalve, Laxe, Bernabeu Guitart, Vilarrasa, & Quemada, 2014). Second, the use of extensive language in the MMSE can lead to unreliable results in aphasic patients and patients who do not speak the local language (Tombaugh & McIntyre, 1992). Another possible limitation is the participation rate of nursing homes and professional caregivers, which might limit the generalizability of the findings if the actual rate might be low.

Patients with ABI will benefit from this study. A recent review about experiences of giving and receiving care in TBI showed that NPS hindered the provision of quality care and required the implementation of proactive nursing strategies to maintain safety for both patients with TBI and nurses (Kivunja, River, & Gullick, 2018). Provision of quality care may be enhanced, e.g., by giving nursing home staff, who are responsible for the daily care of patients with ABI, the tools to improve handling NPS through educational programs. Insight into the magnitude and severity of NPS could give direction to the kind of education that is needed (e.g., education about aggression and ISB). It will also enlarge the awareness of NPS, e.g., apathy, in patients with ABI among physicians, nursing staff and other care professionals. Apathy hardly causes distress according to an explorative study into the relationship between distress and individual NPS of people with dementia in nursing homes (Zwijsen et al., 2014). This could lead to nursing home staff not feeling the urgency to explore this symptom further or to call in a physician or psychologist. Yet, apathy appears, for example, as the most significant risk factor for weight loss (Volicer, Frijters, & van der Steen, 2013). Also, knowledge of NPS and prescribing patterns of PDU in nursing homes may lead to further research, such as longitudinal studies, to determine the course of NPS and PDU and effectiveness studies. This may ultimately lead to recommendations for appropriate use of psychotropic drugs to limit adverse effects (e.g., the use of a limited number of medications that are proven effective).

### 4.2 Conclusion

This study is a first step towards enhancing provision of quality care for these patients and will give more detailed information about the prevalence and characteristics of NPS and PDU. Regardless of the cause of ABI, patients with ABI experience lifelong consequences, such as NPS, that have a high impact on them and their environment. Metaphorically, it is mainly a black box. In long‐term care, the focus shifts from causes of ABI to the consequences, such as NPS. Because an increasing number of patients with severe ABI may survive the acute phase of ABI as a result of modern medicine, it is important to shed light on severe long‐term consequences of ABI, such as NPS, PDU and QoL, in this vulnerable group of patients. Insight into the magnitude and severity of NPS and PDU is fundamental to develop appropriate care for patients with ABI in long‐term care.

## CONFLICT OF INTEREST

The authors declare that they have no conflict of interest.

## AUTHORS’ CONTRIBUTIONS

RFK designed the study and wrote the paper. JL initiated and assisted in the design of the study and co‐wrote the paper. OMS co‐designed the study. DG assisted in the design of the study and co‐wrote the paper. RK assisted in the design of the study and co‐wrote the paper. All authors have been involved in revising the manuscript of the paper and have given final approval for the publication of the paper.
